# A Novel Damage Indicator Based on the Electromechanical Impedance Principle for Structural Damage Identification

**DOI:** 10.3390/s18072199

**Published:** 2018-07-08

**Authors:** Pin Zhou, Dansheng Wang, Hongping Zhu

**Affiliations:** School of Civil Engineering and Mechanics, Huazhong University of Science and Technology, Wuhan, 430074, China; pinzhou1202@hust.edu.cn (P.Z.); hpzhu@hust.edu.cn (H.Z.)

**Keywords:** structural damage identification, lead zirconate titanate (PZT), fourth-order voltage statistical moment (FVSM), beam structure, differential evolution algorithm (DEA), electromechanical impedance (EMI)

## Abstract

This paper presents a novel structural damage detection indicator, i.e., fourth-order voltage statistical moment (FVSM) based on the electromechanical impedance (EMI) principle, and then proposes a two-step damage detection method based on the novel indicator and a differential evolution algorithm (DEA). In this study, several lead zirconate titanate (PZT) sensors bonded to an experimental steel beam were utilized to acquire the time-domain voltage responses. On this basis, the fourth-order voltage statistical moments (FVSMs) of the voltage responses are computed to locate the damage element in the detected structure, and the proposed damage detection method is utilized to quantify the damage. In addition, theoretical PZT voltage responses are also calculated based on the piezoelectric theory and the spectral element method (SEM). Experimental results verify the accuracy of the theoretical voltage values and the effectiveness of the proposed damage indicator. Results indicate that the FVSM is effective in locating the damage element. Integrated with DEA, the proposed technique is capable of quantifying damage.

## 1. Introduction

In the areas of civil, aerospace, and mechanical engineering, structures and their important components must satisfy security criteria throughout their required design lifetime. Therefore, structural damage (even minor initial damage) induced by overloading, fatigue loading, and hostile environments need to be detected timely to ensure the security of the structures. As a non-destructive and real-time structural health monitoring (SHM) technique, the electromechanical impedance (EMI) technique has received much attention recently. It integrates piezoelectric materials such as lead zirconate titanate (PZT) into structures and holds the merits of high frequency actuation and good sensitivity to incipient structural damage [1,2,3,4,5,6,7,8].

The basic principle of the EMI technique is by monitoring the variations of electrical admittance (inverse of impedance) of PZT surface-bonded to or embedded inside the detected structure to determine the presence of structural damage [9,10]. This is due to the variations of the electrical admittance of PZT directly reflects the change of structural mechanical impedance. Root-mean-square deviation (RMSD) is a traditional damage indicator utilized to quantify the variations of admittance within a suggested frequency range from 30 to 400 kHz [11,12,13]. However, determining the location and severity of damage is difficult using RMSD in the applications of the EMI technique [14,15]. This is because the EMI technique is not based on structural mechanical model and the RMSD of the electrical admittances in the frequency domain does not contain the information of damage position. In addition, Lim and Soh [3] also conducted studies to evaluate the feasibility of fatigue crack detection with the RMSD and CCDM indicators based on the EMI method. It is found that the RMSD values fluctuate with the increase of cycles and show no regular pattern. To improve the use of RMSD in the EMI technique, the authors of [16,17,18] applied a sub-frequency interval approach for impedance-based SHM to enlarge the frequency range investigated and eliminate the inconsistency of the RMSD results.

Though researchers mainly focused on monitoring the frequency-domain admittance or impedance when the EMI was used for SHM, there are several investigations that utilized the time-domain impedance signatures. Filho et al. [19] proposed a time-domain analysis approach using wavelet transform to experimentally demonstrate that the time-domain impedance is more sensitivity to structural damage than the frequency-domain one. Silva et al. [20] applied a novel time series analysis for impedance-based SHM. In their study, a random white noise with an amplitude of 1 V was used as the excitation signal, instead of the conventional sine or sine sweep signal. Experimental results in a limited frequency range of 25–45 kHz verified the feasibility of the proposed SHM method. Typically, commercial impedance analyzers such as HP4194A and Agilent4294A are generally used in the EMI technique to realize the impedance signal collection due to the merits of high measurement accuracy. However, the bulkiness and the high cost of commercial impedance analyzers restrict the application of the EMI technique in on-site SHM [21,22,23]. In addition, it is difficult to measure the time-domain impedance signatures by using the commercial impedance analyzers. To broaden the availability of the EMI technique for SHM, it is essential to develop a low-cost and portable impedance measurement system. Xu and Giurgiutiu [24] designed an impedance testing system, in which a function generator was adopted to provide the excitation signal and a data acquisition device was used to sample the excitation and response signals. Cortez et al. [25] developed a micro-controlled SHM system assembled from an evaluation board, a direct digital synthesizer, and a low-pass filter. By using the SHM system, the root mean square voltage rather than the conventional electrical impedance of PZT were monitored to detect structural damage. The foregoing impedance testing systems are highly dependent on the integrated circuits and associated electrical knowledge. Moreover, wireless systems [26,27] with the AD5933 impedance measurement chip have also been researched by various authors to manage the EMI technique when monitoring large-scale structures. With these wireless systems, the cost and bulkiness problem of commercial impedance analyzer can be also solved. More conveniently, AD5933 Evaluation Board’ was commercialized by Analog Devices, Inc. (Norwood, MA, USA). However, the board can only measure impedance up to 100 kHz. Convenient for the use by the engineers, Wang and Li [28] established an impedance measurement system containing only a signal generator and an oscilloscope for testing the dual-PZT type transducer. It holds the merits of a low-cost and portable and can measure the time-domain impedance signatures.

To improve the anti-noise performance, a damage index based on statistical moment has been studied recently. Zhang et al. [29] and Xu et al. [30] proposed the displacement statistical moment as the damage indicator to detect structural damage, and results confirmed that the proposed damage indicator is insensitive to ambient noise but sensitive to local structural damage. Xiang et al. [31] and Wang et al. [32,33,34] proposed the strain statistical moment and applied it to beam-type and plate structures for damage detection. Numerical and experimental results demonstrated that the strain statistical moment is feasible for detecting incipient structural damage and insensitive to measurement noise. Alamdari et al. [35] recently also proposed a new non-model-based spectral moment method to detect the damage of the jack arches in a bridge structure by combining a modified k-means−clustering algorithm. They pointed out that the statistical moments contain information from the entire frequency range, which enables the detection of subtle differences between the normal signals and distorted ones.

The structural damage identification techniques based on the finite element model updating have made great progress. However, the damage identification results are highly dependent on the mesh size and the number of finite elements. The spectral element method (SEM) has proven a precise calculation method and requires a smaller number of elements than FEM for calculating the structural responses [36]. Lee et al. [37,38] used SEM to obtain the dynamic responses of one-dimensional structures and Levy-type plates. Krawczuk [39] successfully identified beam damage by using the proposed damage detection method based on the SEM and a genetic algorithm (GA). Wang et al. [40,41] also established a damage spectral element model to simulate structural wave propagation and global vibration properties for damage identification. With the help of the spectral element model updating, the crack damage in a steel beam was detected successfully. Sun et al. [42,43] simulated the impedance signals based on the Fourier transform-based spectral element model and identified debonding damage in FRP strengthened concrete beams with the combination of particle swarm optimization (PSO) algorithm.

In this paper, a novel damage detection indicator called the fourth-order voltage statistical moment (FVSM) is presented to avoid the trial-and-error of the frequency range selection in the EMI technique and to quantify the structural damage. The measurement system established by Wang and Li [28] is employed to conveniently measure the time-domain electrical response of PZT. The voltage responses of the PZT surface-bonded to the detected structure is first theoretically derived and compared with the experimental results. The FVSM indicator is then used to locate the damage element of an experimental steel beam. Combined with a differential evolution algorithm (DEA), the proposed damage detection method is utilized to quantify the damage of the experimental beam.

## 2. Fourth Voltage Statistical Moment-Based Damage Detection Method

### 2.1. Spectral Element Formulation for a Timoshenko Beam

#### 2.1.1. The Intact Beam

For a Timoshenko beam, the governing differential equations can be expressed as

(1) $$EA\frac{\partial^{2}u}{\partial x^{2}}-\rho A\frac{\partial^{2}u}{\partial t^{2}}=0$$

(2) $$\kappa GA\left(\frac{\partial^{2}v}{\partial x^{2}}-\frac{\partial \varphi }{\partial x}\right)=\rho A\frac{\partial^{2}v}{\partial t^{2}}$$

(3) $$EI_{z}\frac{\partial^{2}\varphi }{\partial x^{2}}+\kappa GA\left(\frac{\partial v}{\partial x}-\varphi \right)=\rho I_{z}\frac{\partial^{2}\varphi }{\partial t^{2}}$$

where *u* is the axial displacement; *v* and *ϕ* are the shear and rotational displacements, respectively; *E* and *G* denote the Young’s modulus and shear modulus, respectively, and *G* = *E*/2(1 + *μ*); *κ* = (0.87 + 1.12*μ*)^2^/(1 + *μ*)^2^ is the Timoshenko shear coefficient; *A*, *ρ*, and *I_z_* are the cross-sectional area, density, and area moment of inertia, respectively.

Laplace transform (LT) is applied to both sides of Equations (1)–(3). Then, similar to [44], with the general solution and the mechanical and displacement boundary conditions of Timoshenko beam, the dynamic stiffness matrix of spectral element can be obtained as

(4) $$K_{e}=B_{e}D_{e}{}^{-1}$$

where **K***_e_* is the dynamic stiffness matrix. **B***_e_* and **D***_e_* are the spectral element force and spectral element displacement matrices, respectively.

(5) $$B_{e}=\left[\begin{array}{cccccc} -ik_{3}EA & ik_{3}EAb & 0 & 0 & 0 & 0\\ 0 & 0 & -\varepsilon (ik_{1}+\alpha_{1}) & \varepsilon (ik_{1}+\alpha_{1})p & -\varepsilon (ik_{2}+\alpha_{2}) & \varepsilon (ik_{2}+\alpha_{2})a\\ 0 & 0 & -ik_{1}\alpha_{1}EI_{z} & -ik_{1}\alpha_{1}EI_{z}p & -ik_{2}\alpha_{2}EI_{z} & -ik_{2}\alpha_{2}EI_{z}a\\ -ik_{3}EAb & ik_{3}EA & 0 & 0 & 0 & 0\\ 0 & 0 & -\varepsilon (ik_{1}+\alpha_{1})p & \varepsilon (ik_{1}+\alpha_{1}) & -\varepsilon (ik_{2}+\alpha_{2})a & \varepsilon (ik_{2}+\alpha_{2})\\ 0 & 0 & -ik_{1}\alpha_{1}EI_{z}p & -ik_{1}\alpha_{1}EI_{z} & -ik_{2}\alpha_{2}EI_{z}a & -ik_{2}\alpha_{2}EI_{z} \end{array}\right]$$

(6) $$D_{e}=\left[\begin{array}{cccccc} 1 & b & 0 & 0 & 0 & 0\\ 0 & 0 & 1 & p & 1 & a\\ 0 & 0 & \alpha_{1} & -\alpha_{1}p & \alpha_{2} & -\alpha_{2}a\\ b & 1 & 0 & 0 & 0 & 0\\ 0 & 0 & p & 1 & a & 1\\ 0 & 0 & \alpha_{1}p & -\alpha_{1} & \alpha_{2}a & -\alpha_{2} \end{array}\right]$$

where $k_{3}=is\sqrt{\rho /E}$ is the wave number of the stretching mode of the beam; both $k_{1}=\sqrt{(x_{1}+\sqrt{x_{1}^{2}-4x_{2}})/2}$ and $k_{2}=\sqrt{(x_{1}-\sqrt{x_{1}^{2}-4x_{2}})/2}$ are the wave numbers of the bending mode of the beam; $x_{1}=-\rho s^{2}\left[1/\kappa G+1/E\right]$, $x_{2}=\rho^{2}s^{4}/\kappa EG+\rho As^{2}/EI_{z}$, and $\alpha_{j}=(k_{j}^{2}\kappa G+\rho s^{2})/ik_{j}\kappa G,\;j=1,\;2$; *s* = *iw* + *σ* is the Laplace transform parameter; *w* is the circular frequency; *i* is the complex unit; σ=2π/(NΔt), where *N* and Δ*t* are sampling point number and time interval in the Laplace transform, respectively; ε=κGA, $p=e^{-ik_{1}L}$, $a=e^{-ik_{2}L}$; $b=e^{-ik_{3}L}$; *L* is the length of the beam element.

#### 2.1.2. The Cracked Beam

**Figure 1:**
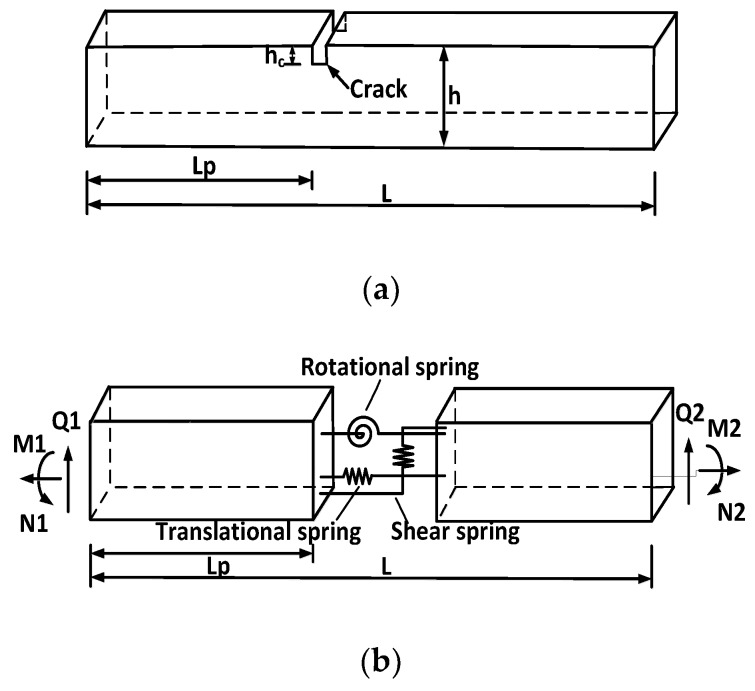
(**a**) Cracked beam segment and (**b**) its spectral beam element model.

The spectral element formulation for a cracked Timoshenko beam is similar to that for the intact one. Figure 1 shows a spectral beam element model of the cracked beam segment. *L_p_* and *h_c_* are the location and depth of the crack in the cracked beam element, respectively. *h* is the beam height. *N_j_*, *Q_j_*, and *M_j_* (*j* = 1, 2) denote the axial forces, shear forces, and bending moments at the ends of the cracked beam element, respectively. For a cracked spectral element, a translational spring, a rotational spring, and a shear spring are introduced simultaneously to simulate the opening and non-propagating crack. To obtain the spectral element stiffness matrix of a cracked beam element, the most important step is to obtain the general solutions for the two components of the beam element divided by the crack in the same local coordinate system and then to establish the relationship between the node force and the node displacement based on the continuity conditions at the crack position. The deduction details can be found in [45]. The dynamic stiffness matrix for a cracked Timoshenko beam element is given:

(7) $$K_{ec}=B_{ec}D_{ec}^{-1}$$

where the subscript *c* denotes the cracked beam element. **B***_ec_* and **D***_ec_* are the spectral crack element force and spectral crack element displacement matrices, respectively. Their specific formulations can be found in [45]. It is noted that **B***_ec_* and **D***_ec_* are obtained by LT in this paper other than by Fourier transform in [45].

The global dynamic stiffness matrices of the intact and cracked beams can be obtained by assembling the corresponding element stiffness matrices. It is noted that the surface-bonded PZT is not considered in the spectral element model because it is much smaller than the beam structure.

### 2.2. Voltage Responses of the PZT Bonded on a Beam

The exciting voltage on PZT produces axial force *N* and moment *M* to the base beam. Neglecting the effect of the bonding layer, the expressions for force *N* and moment *M* are achieved:

(8) $$N=b_{p}d_{31}Y_{11}V$$

(9) $$M=b_{p}d_{31}Y_{11}^{}(\frac{h_{b}}{2}+\frac{h_{p}}{2})V$$

where *h_b_* is the height of the base beam; *b_p_*, *h_p_*, *d*_31_, and *Y*_11_ are the width, height, piezoelectric strain constant, and Young’s modulus of the PZT, respectively; *V* is the voltage imposed on PZT in the frequency domain. The dynamic responses of the base beam can be deduced from the structural global dynamic stiffness matrix and the forces *N*.

Considering the piezoelectric equations of PZT [9],

(10) $$\begin{array}{l} s_{1}=\frac{1}{Y_{11}^{E}}T_{1}+d_{31}E_{3}\\ D_{3}=d_{31}T_{1}+\varepsilon_{33}^{E}E_{3} \end{array}.$$

Then, eliminating the stress along the beam length direction *T*_1_, the electric displacement *D*_3_ of PZT can be expressed as

(11) $$D_{3}=d_{31}Y_{11}^{E}s_{1}+(\varepsilon_{33}^{E}-d_{31}^{2}Y_{11}^{E})E_{3}$$

where *E*_3_ is the electric field strength; $Y_{11}^{E}=Y_{11}(1+\eta i)$ is the complex Young’s modulus of PZT at the zero electric field; *η* is the mechanical loss factor; $\varepsilon_{33}^{E}=\varepsilon_{33}(1-\delta i)$ is the complex dielectric constant at zero stress; *δ* is the dielectric loss factor.

Then, the electric current of PZT can be obtained as

(12) $$\begin{array}{ll} I & =s\iint D_{3}dA\\ & =sd_{31}Y_{11}^{E}b_{p}[\bar{u}-(h+h_{p})/2\bar{\varphi }]+sb_{p}l_{p}(\varepsilon_{33}^{E}-d_{31}^{2}Y_{11}^{E})V/h_{p} \end{array}$$

where $\bar{u}$ and $\bar{\varphi }$ denote the difference of axial displacements and rotation angles between the two ends of the PZT bonded on the base beam, respectively. $\bar{u}$ and $\bar{\varphi }$ can be obtained from the above-mentioned SEM model of the beam.

With the electric current *I* transformed into the time domain *I*(*t*) through inverse LT, the time-domain voltage response of the PZT *U* is achieved:

(13) $$U=V(t)-I(t)R_{c}$$

where *R_c_* is the resistor value.

### 2.3. Theory of Statistical Moment

When a linear structure system is subjected to a stationary Gaussian random process, the structural response will also be a stationary Gaussian random process [31]. For the Gaussian distribution, the response’s probability density function and different order statistical moment can be expressed as

(14) $$p(r)=\frac{1}{\sqrt{2\pi \sigma }}e^{\frac{-{\left(r-\bar{r}\right)}^{2}}{2\sigma^{2}}}$$

(15) $$M_{n}={\int_{-\infty }^{+\infty }\left(r-\bar{r}\right)}^{n}p(r)dr\;n=1,2,3,4\ldots \ldots$$

where *r* is the structural response; *p*(*r*) is the probability density function of *r*; $\bar{r}$ and *σ* are the mean value and the variance of *r*, respectively; *M_n_* is the nth order statistical moment of *r*.

Taking the sensitivity and stability of the statistical moment into account in the damage detection method, previous studies [30,31,32,33,34] selected the fourth-order moment as the damage indicator even though a higher-order moment has better sensitivity to structural damage. Furthermore, whether the structural response obeys Gaussian random distribution or not, the following equation can be used to calculate the fourth-order statistical moment:

(16) $$M_{4}=\frac{1}{N}\sum_{i=1}^{N}r_{i}^{4}-\frac{4}{N}\bar{r}\sum_{i=1}^{N}r_{i}^{3}+\frac{6}{N}{\bar{r}}^{2}\sum_{i=1}^{N}r_{i}^{2}-3{\bar{r}}^{4}$$

where *N* is the number of sampling points of *r*.

### 2.4. Differential Evolution Algorithm

Storn and Price [46,47] first applied DEA to solve optimization problems in the 1990s. In the International Competition on Evolutionary Optimization held in Indianapolis, the DEA was shown to be a fast evolutionary algorithm [48]. The framework of DEA is similar to that of GA, that is, it includes initialization, mutation, crossover, selection and convergence. Their difference is that binary digits are substituted by real floating point numbers in the same way when crossover and mutation are executed. Moreover, the advantages of DEA include ease of implementation, minimal parameter tuning and good astringency.

For DEA, the assigned value of the population size (AP), the differential weight (DW), and the crossover factor (CR) are of great significance for improving convergence and increasing offspring diversity. An appropriate AP guarantees that the feasible parameter space is absolutely spanned. On the one hand, a too-small AP results in unsatisfactory sampling of the population in the search space. On the other hand, an excessively large AP is not good for convergence. Storn and Price [46] suggested that AP is chosen between 5 and 10 times the number of the optimization parameters. In the step of mutation, DW controls the magnification of the differential variation, and the constant is recommended to be chosen within the range from 0.5 to 1. For the last control variable CR, a reasonable initial value of 0.1 or 0.9 is advised.

### 2.5. Two-Step Damage Detection Method

**Figure 2:**
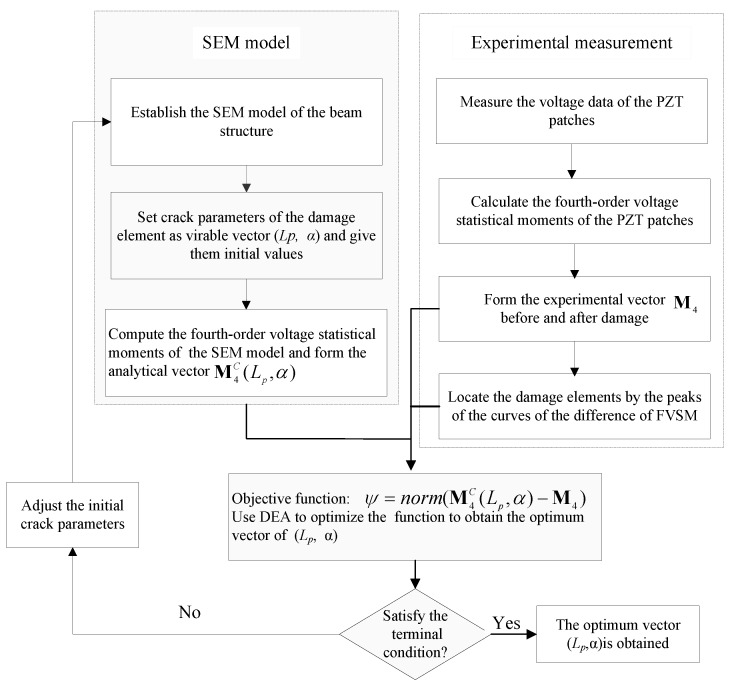
Flowchart of the proposed damage detection method.

The basic idea of the FVSM-based damage detection method in the time domain is that the voltage responses of PZT can reflect the health status of the host structure. Therefore, the presence of damage in the structure will cause non-negligible changes in voltage responses of PZT. Furthermore, in the data processing, the statistical moment not only weakens the impact of measurement noise and reduces the calculation cost but also enlarges the frequency range used in structural damage detection. The objective of this study is to locate the damaged spectral element and quantify the damage size. A flowchart of the proposed damage detection method is shown in Figure 2, and the detailed procedure is described as follows.

The voltage responses of each PZT surface-bonded on the intact beam are first measured and the time-domain voltage of the mth PZT is denoted as $U_{m}=[U_{m1},U_{m2},\ldots \ldots ,U_{mN}]$. According to Equation (16), the FVSM *M*_4*m*_ of the voltage response can be calculated as

(17) $$M_{4m}=\frac{1}{N}\sum_{n=1}^{N}U_{mn}{}^{4}-\frac{4}{N}{\bar{U}}_{m}\sum_{n=1}^{N}U_{mn}{}^{3}+\frac{6}{N}{\bar{U}}_{m}{}^{2}\sum_{n=1}^{N}U_{mn}{}^{2}-3{\bar{U}}_{m}{}^{4}.$$

Therefore, the FVSMs of all PZT patches surface-bonded on the beam can be calculated and denoted as vector *M*_4_. When damage occurs in the structure, *α* denotes the ratio of the depth of crack to the height of the beam. To locate the damage element, the FVSM difference before and after damage of the beam will be obtained. Then, the damage element can be ascertained through finding the curve peak of the FVSM difference. The analytical vector $M_{4}^{C}(L_{p},\alpha )$ obtained from the spectral element model of the beam can also be established. Combined with the DEA, the objective function expressed as Equation (18) is optimized to identify the specific damage element and damage severity.

(18) $$\psi =\mathrm{norm}(M_{4}^{C}(L_{p},\alpha )-M_{4})$$

where norm(.) returns the Euclidean distance of the input vector.

## 3. Experimental Setup

**Figure 3:**
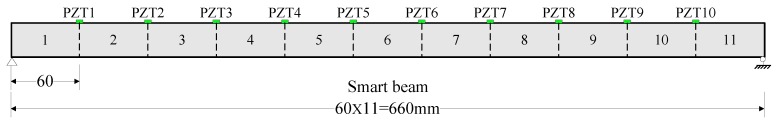
Simply supported beam.

To assess the effectiveness of the proposed FVSM-based damage detection method in the time domain, experiments were conducted on a simply supported steel beam with a length of 660 mm, a width of 37.8 mm, and a thickness of 5.6 mm, as shown in Figure 3. The modulus of elasticity, density, and Poisson’s ratio of the beam were 2.1 × 10^11^ N/m^2^, 7800 kg/m^3^, and 0.3, respectively. The beam was divided into 11 elements, and 10 PZT patches with the dimension of 10 × 10 × 0.5 mm^3^ were uniformly surface-bonded on the beam. The distance between any two adjacent PZT patches was 60 mm. Considering Timoshenko beam theory, each node had three degrees of freedom. The detailed geometric and material parameters of the simply supported beam model and the PZT patches are listed in Table 1 and Table 2, respectively.

**Figure 4:**
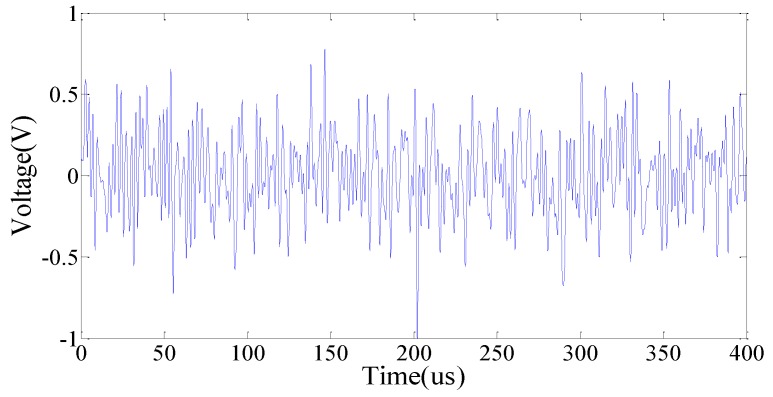
Voltage excitation signal.

**Figure 5:**
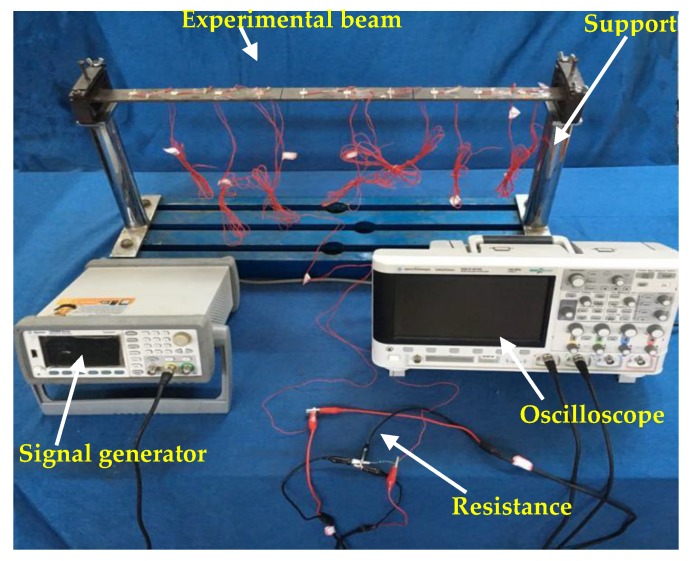
Experimental arrangement.

In this experiment, a Gauss white noise (GWN) excitation of 400 microseconds duration, a 1 V amplitude, and a frequency range from 0 to 500 kHz was simulated by MATLAB, as shown in Figure 4. The GWN signal was imposed using a signal generator (Agilent 33522B) to generate the expected excitation to the PZT patches. It is necessary to mention that the amplitude of the GWN signal was modulated to 10 Vpp in the signal generator before trigger during the experiment. Two kinds of damage, i.e., the added mass and the crack, were artificially introduced. An oscilloscope (Agilent DSOX2014A) was used to capture the voltage responses of all the PZT patches. A fixed-value resistance (*R_c_* = 47 Ω) was connected in series with the detected part. Figure 5 shows the experimental arrangement.

In the mass added experiment, masses of 10, 20, 50, 100, and 200 g were added to the middle of the eighth element, respectively. They have been denoted as Cases 1 to 5 (D1, D2, D3, D4, and D5) in turn. Two dual-damage cases (D6 and D7) were also preset by simultaneously adding mass of 50 or 200 g to the middle of the fifth and eighth elements.

**Figure 6:**
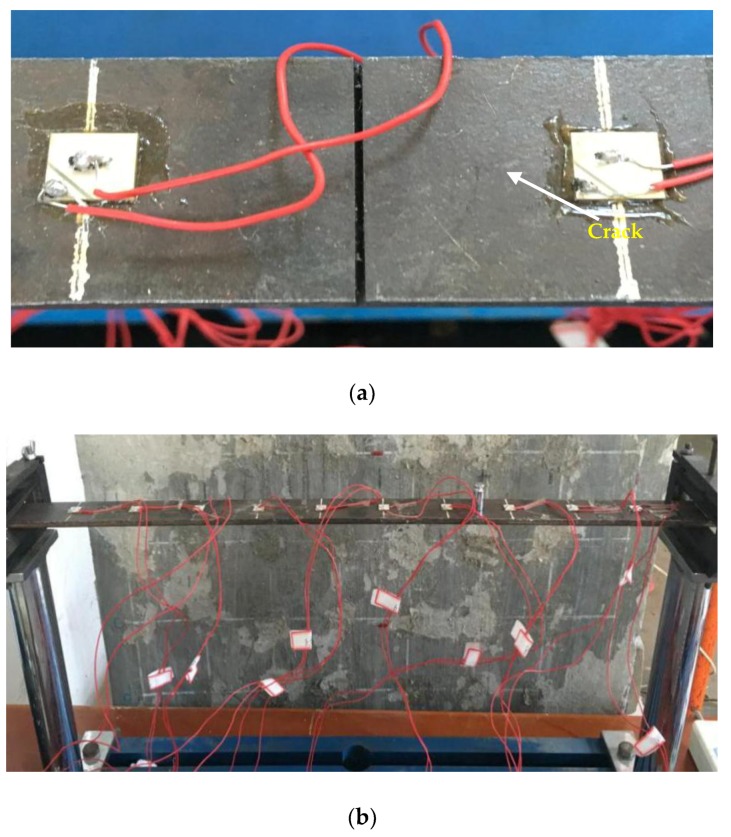
Representative damage for the experimental beam: (**a**) S3 and (**b**) D1.

In the crack detection experiment, crack damage was introduced to the experimental beam. Three damage cases were preset by making a 1-mm-wide through crack along the width of the beam with a wire cut machine. The crack was located at the middle of the eighth element. The 1-mm-deep crack is denoted as Damage Case 1 (S1), the 2-mm-deep crack is denoted as Damage Case 2 (S2), and the 3-mm-deep crack is denoted as Damage Case 3 (S3). Figure 6 shows the introduced damage. The details of the preset cases for the simply supported beam are listed in Table 3. In addition, to guarantee the measurement accuracy, the experiment was conducted over a short period of time, and the temperature was relatively stable, about 13 °C.

## 4. Experimental Results and Analysis

### 4.1. Comparison between the Experimental and Analytical Voltage Responses

**Figure 7:**
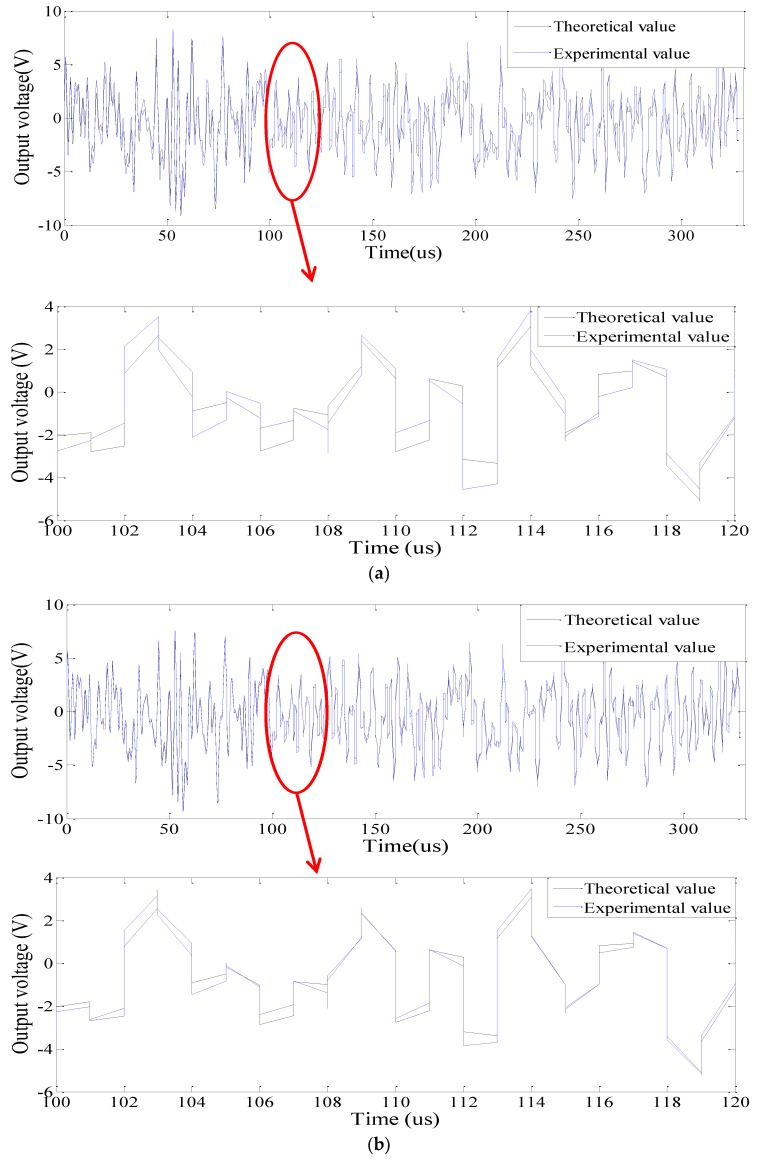
Output voltage signals of representative PZT patches for the intact beam: (**a**) PZT2; (**b**) PZT5.

With the developed measurement system, the experimental voltage response signals of PZT1–PZT10 for the intact beam case were measured, and the results were compared with the analytical results based on the SEM. Figure 7 shows the comparison of two representative PZT patches: PZT2 and PZT5. The experimental results of all PZT patches have good consistency with the theoretical ones. The correlation coefficients between the experimental and theoretical voltage curves were also calculated by function of corrcoef(.) in MATLAB to quantitatively valuate their consistency. The correlation coefficient values of each PZT patch are listed in Table 4.

### 4.2. Damage Element Location in the Added Mass Experiment

**Figure 8:**
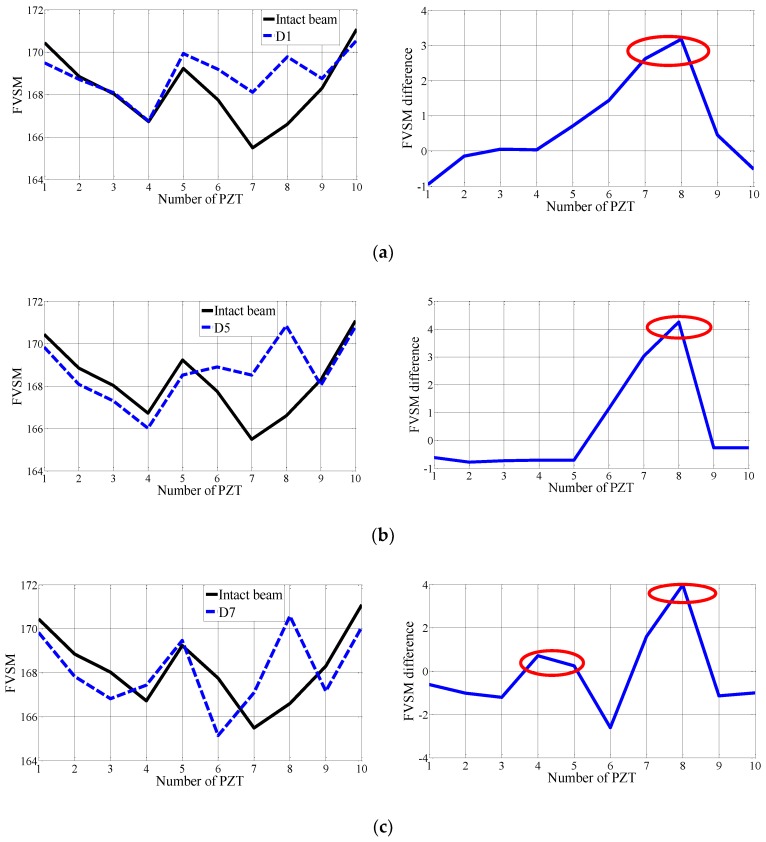
Representative FVSM and FVSM difference curves for the experimental beam in different added mass cases compared with the intact case: (**a**) D1; (**b**) D5; (**c**) D7.

**Figure 9:**
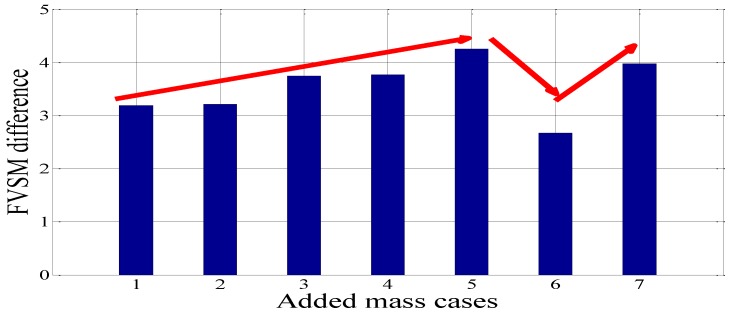
FVSM difference values for PZT 8 in the added mass cases compared with the intact case.

In this experiment, the FVSM-based damage detection technique was used to detect the artificial damage induced by adding mass to the experimental beam. The voltage responses of all the 10 PZT patches were first measured for the undamaged beam. The voltage responses for seven added mass cases, D1–D7, were then acquired step by step. With the use of Equation (19), the FVSM values of all the PZT patches were calculated for the beam before and after mass was added. The FVSM differences were obtained for damage detection. In all FVSM difference curves for Cases D1–D7, apparent peaks were observed at the added mass elements. Typical FVSM and FVSM difference curves for added mass cases D1, D5, and D7 are shown in Figure 8. Notably, a relationship exists between the magnitude of the FVSM difference of the added mass element and the mass quantity. In Cases D1 to D5, with the increases of the mass added to the eighth element, the FVSM difference values of the added mass element show an increasing tendency from 3.183 to 4.25. In Cases D6 and D7, the FVSM difference values of the eighth element are 2.667 and 3.975, and an increasing tendency is also apparent, as shown in Figure 9. It should be noted that the FVSM difference value of the eighth element in Case D6 is smaller than that in Case D3. This is because the added mass is added to the fifth element, whose influence on the simply supported beam is smaller than that of the mass added to the eight element. The same phenomenon appears in Case 7. In sum, the FVSM difference is sensitive to single and multiple added masses.

### 4.3. Damage Element Location in Crack Damage Experiment

**Figure 10:**
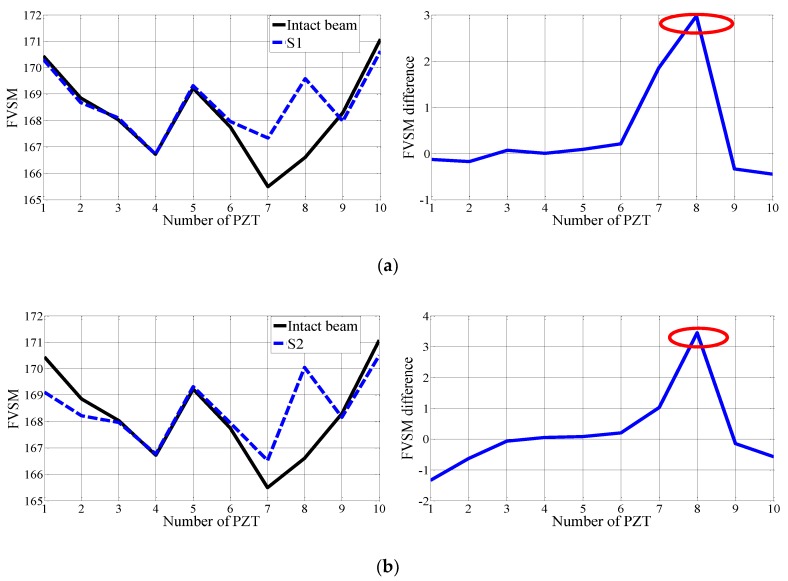

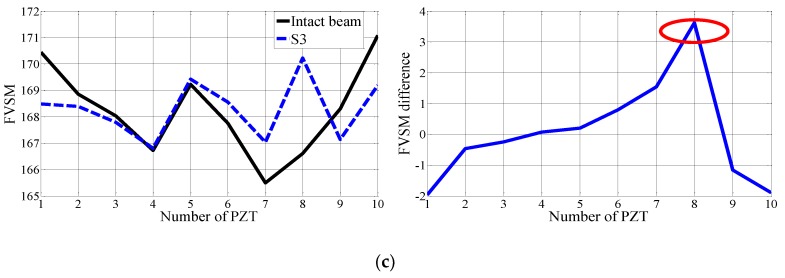
FVSM and FVSM difference curves for the experimental beam in different crack damage cases compared with the intact case: (**a**) S1; (**b**) S2; (**c**) S3.

**Figure 11:**
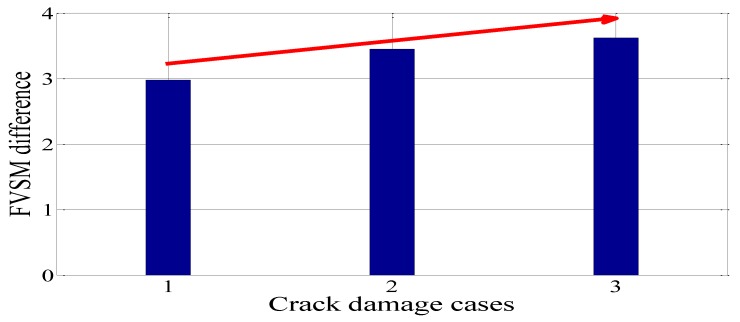
FVSM difference values for PZT 8 in the crack damage cases compared with the intact case.

In this experiment, the FVSM-based damage detection technique was used to detect the crack damage of the experimental beam. The voltage responses of all 10 PZT patches were measured in the undamaged case and the three crack damage cases S1–S3. Following the same calculation process described in Section 4.2, Figure 10 shows the FVSM and FVSM difference curves in each damage case. In the FVSM difference curves for Damage Cases S1–S3, apparent peaks were also observed at the damage elements. The same finding was observed; namely, the magnitude of the FVSM difference of the damage element increases with the increase of crack depth. For Damage Cases S1–S3, Figure 11 shows that the FVSM difference values of the damage element increase from 2.978 to 3.618 as crack depth increases. The identification results show that it is feasible to locate crack damage using the FVSM difference.

It was also found that it is difficult to directly locate beam damage using the blue lines of the FVSM curve from Figure 8 and Figure 10. This indicates that the proposed damage identification method needs the data in a structural health state as the baselines to obtain an FVSM difference curve that locates beam damage.

### 4.4. Damage Quantification Based on the SEM and DEA

In the prior section, beam damage can be localized at certain or several spectral elements using the proposed FVSM indicator. Here, the damage quantification was implemented by utilizing the proposed damage detection method in Section 2.5. In this study, without loss of generality, only crack damage was quantified by the combination of the SEM and DEA method. For each crack damage case, the baseline data were denoted as matrix assembled by the FVSM values calculated from the voltage responses of all PZT patches. The distance between the crack and the left end of the damage element *L_p_* was used as an optimization parameter to locate the damage, and the other optimization parameter *α* that represents the ratio of the crack depth to the beam height was used for damage quantification. With an initial value assigned to the optimization parameter vector, a model updating procedure based on the DEA algorithm and the SEM was implemented to identify the location and depth of the damage simultaneously.

For the DEA, to improve the convergence and increase the offspring diversity. population size AP was set to 12, differential weight DW set to 0.5, and crossover factor CR to 0.9. The identified Lp and *α* in all the damage cases are listed in Table 5, showing that the damage locations and severities can be identified. The maximal identification errors are 16.7% for damage location and 10.5% for damage severity. It was found that the time cost of the updating method for damage quantification is about 5000 s, which was calculated from an ordinary personal computer with an Intel Core i7 920@2.67GHz CPU and a 4 GB memory. It is clear that the proposed novel damage indicator combined with the SEM and the DEA is capable of localizing and quantifying the beam damage despite some identification errors. In the future, we will further modify the optimization algorithm to improve the damage identification accuracy.

## 5. Conclusions

In this paper, a novel damage detection indicator (the FVSM) is proposed to detect the damage of a steel beam. Integrated with the Laplace-based SEM and the DEA algorithm, a two-step damage detection technique was developed to locate and quantify the beam damage. Experiments for the steel beam were conducted, bringing in an added mass element and a cracked element. Experimental results demonstrate the effectiveness and sensitivity of the proposed damage indicator. Theoretical PZT voltage responses are also calculated based on the piezoelectric theory and the spectral element method (SEM). Experimental results verify the accuracy of the theoretical voltage values, too. The FVSM difference effectively locates the added mass element or the cracked element of the beam. The proposed two-step damage detection technique is capable of localizing and quantifying the beam damage with acceptable accuracy.

## Figures and Tables

**Table 1:** Relevant parameters of the simply supported beam.

Geometrical Parameters	Value	Physical Parameters	Value
Length (mm)	660	Elastic modulus (Pa)	2.1 × 10^11^
Width of section (mm)	37.8	Poisson’s ratio	0.3
Height of section (mm)	5.6	Density (kg/m^3^)	7800
Section type	Rectangle		

**Table 2:** Dimensions and material properties of PZT patches.

Symbol	Name	Value
$l_{p}$	Length	10 mm
$b_{p}$	Width	10 mm
$h_{p}$	Thickness	0.5 mm
$\rho_{p}$	Density	7860 kg/m^3^
$Y_{11}$	Young’s modulus	60.16 GPa
η	Mechanical loss factor	0.0005
$\varepsilon_{33}$	Dielectric constant	1.311 × 10^−8^ F/m
δ	Dielectric loss factor	0.025
$d_{31}$	Piezoelectric constant	−1.43 × 10^−10^ m/V

**Table 3:** Details of cases for the simply supported beam.

Cases	Element 5	Element 8	Cases	Element 8
D1	–	10 g	S1	1 mm cut
D2	–	20 g	S2	2 mm cut
D3	–	50 g	S3	3 mm cut
D4	–	100 g		
D5	–	200 g		
D6	50 g	50 g		
D7	200 g	200 g			

**Table 4:** Correlation coefficient values between the analytical and experimental voltage signals of each lead zirconate titanate (PZT) patch for the intact beam.

PZT1	PZT2	PZT3	PZT4	PZT5	PZT6	PZT7	PZT8	PZT9	PZT10
0.916	0.964	0.989	0.995	0.991	0.996	0.982	0.986	0.978	0.984

**Table 5:** Identified damage results and relative errors for the experimental beam.

Damage Cases	Preset Damage (*L_p_*, *α*)	Identified Damage (*L_p_*, *α*)	Relative Error (%)	Time Cost (s)
S1	0.03, 0.1786	0.025, 0.1742	16.7, 2.5	4898
S2	0.03, 0.3571	0.028, 0.3194	6.7, 10.5	5032
S3	0.03, 0.5357	0.028, 0.5018	6.7, 6.3	5136
